# Control of Established Colon Cancer Xenografts Using a Novel Humanized Single Chain Antibody-Streptococcal Superantigen Fusion Protein Targeting the 5T4 Oncofetal Antigen

**DOI:** 10.1371/journal.pone.0095200

**Published:** 2014-04-15

**Authors:** Kelcey G. Patterson, Jennifer L. Dixon Pittaro, Peter S. Bastedo, David A. Hess, S. M. Mansour Haeryfar, John K. McCormick

**Affiliations:** 1 Department of Microbiology and Immunology, Western University, London, Ontario, Canada; 2 Centre for Human Immunology, Western University, London, Ontario, Canada; 3 Department of Physiology and Pharmacology, Western University, London Ontario, Canada; 4 Vascular Biology Research Group, Robarts Research Institute, London, Ontario, Canada; 5 Lawson Health Research Institute, London, Ontario, Canada; Carl-Gustav Carus Technical University-Dresden, Germany

## Abstract

Superantigens (SAgs) are microbial toxins that cross-link T cell receptors with major histocompatibility class II (MHC-II) molecules leading to the activation of large numbers of T cells. Herein, we describe the development and preclinical testing of a novel tumor-targeted SAg (TTS) therapeutic built using the streptococcal pyrogenic exotoxin C (SpeC) SAg and targeting cancer cells expressing the 5T4 tumor-associated antigen (TAA). To inhibit potentially harmful widespread immune cell activation, a SpeC mutation within the high-affinity MHC-II binding interface was generated (SpeC_D203A_) that demonstrated a pronounced reduction in mitogenic activity, yet this mutant could still induce immune cell-mediated cancer cell death *in vitro*. To target 5T4^+^ cancer cells, we engineered a humanized single chain variable fragment (scFv) antibody to recognize 5T4 (scFv5T4). Specific targeting of scFv5T4 was verified. SpeC_D203A_ fused to scFv5T4 maintained the ability to activate and induce immune cell-mediated cytotoxicity of colorectal cancer cells. Using a xenograft model of established human colon cancer, we demonstrated that the SpeC-based TTS was able to control the growth and spread of large tumors *in vivo*. This required both TAA targeting by scFv5T4 and functional SAg activity. These studies lay the foundation for the development of streptococcal SAgs as ‘next-generation’ TTSs for cancer immunotherapy.

## Introduction

Superantigens (SAgs) are microbial toxins that function as potent T cell activators and are mediators of the toxic shock syndrome [1]. These molecules function by binding to lateral surfaces of major histocompatibility class II (MHC-II) molecules [2]–[5], while simultaneously engaging germline-encoded regions within the variable region of the T cell receptor (TCR) β-chain (Vβ) [6]–[9]. Since there are ∼50 functional Vβ genes in humans [10], [11], and because different SAgs can often target multiple Vβs [12], these toxins stimulate a very large percentage of exposed T cells leading to the subsequent release of pro-inflammatory cytokines (e.g. IL-2, IFN-γ, and TNF-α) [1]. Although SAgs do not engage MHC I molecules, these toxins do activate both CD4^+^ and CD8^+^ T cells [13], and this can subsequently lead to bystander activation of accessory cells including NK cells [14]. In specific cases, SAg can also activate unconventional T cell subsets such as invariant natural killer T (*i*NKT) cells [15] and γδ T cells [16].

The ultimate goal of cancer immunotherapy is to harness immune-mediated mechanisms to specifically target and eradicate tumor cells. There have been significant efforts to design SAg-based immunotoxins, also known as tumor-targeted superantigens (TTS), in order to artificially ‘force’ T cells to recognize tumor-associated antigens (TAAs) in a non-HLA-restricted manner. The initial TTS represented the fusion of a mouse antibody fragment (Fab) targeting a colorectal cancer antigen, to the wild-type staphylococcal enterotoxin A (SEA) SAg. In this pioneering work, the Fab::SEA TTS demonstrated a substantial reduction in tumor burden and mortality using a B16 mouse metastasis model [17]. Later studies utilized the fusion of a mouse Fab to target the 5T4 oncofetal antigen with a mutated version of SEA (designed to reduce MHC-II binding) and this resulted in ∼95% reduction of tumor mass in a non-small cell lung cancer (NSCLC) model [18]. Furthermore, combination therapies with TTSs have also shown promise in preclinical models in conjunction with cytokine therapies (e.g. IFN-α) [19] and blockade of CTLA-4 [20]. These and other studies have clearly demonstrated the potential of TTSs for cancer immunotherapy. Nonetheless, the TTSs have so far been built exclusively using members of the SE class of SAg, and SEs are also agents of staphylococcal food-borne illness, an activity that is thought to be independent of the ability to activate T cells [21]. Although manageable, some of the side effects seen in TTS Phase I and Phase II clinical trials included nausea, vomiting and diarrhea [22]–[24], and may have been related to the emetic properties of SEA [25]. Additionally, many patients had pre-existing antibodies to SEA which required individualized TTS dosing [26]. In order to reduce the antigenicity of the TTS therapeutic, anti-5T4 Fab was linked to an engineered SEA/SEE fusion (called Naptumomab estafenatox; ABR-217620) [27]. This latest TTS therapeutic has undergone a Phase I clinical trial as a monotherapy in patients with advanced NSCLC, pancreatic cancer and renal cell carcinoma (RCC), and as a combination therapy with Docetaxel in patients with NSCLC, demonstrating that ABR-217620 was well tolerated with some evidence of anti-tumor activity [24]. Early information from a recently completed Phase II/III trial with ABR-217620 in patients with RCC comparing ABR-217620 and interferon-α, to interferon-α alone, did not reach the primary endpoint of overall survival; however, it appears that many patients had higher than expected baseline levels of anti-SEA/SEE antibodies, which may have contributed to suboptimal therapy [28].

Bacterial genomic sequencing efforts over the last decade have now revealed an extensive ‘family’ of SAg exotoxins in both *Staphylococcus aureus* and *Streptococcus pyogenes*. A general feature of these toxins is that genetically distinct SAgs are also antigenically distinct, and furthermore, distinct SAgs also typically display unique Vβ activation profiles [12]. Thus, *S. aureus* and *S. pyogenes* have provided an abundance of T cell mitogens that could potentially be engineered as TTSs for cancer therapy. In the current work, we sought to expand the repertoire of TTSs to include the first streptococcal SAg using streptococcal pyrogenic exotoxin C (SpeC) as the prototype. A potential advantage of engineering a streptococcal SAg as a TTS is that these toxins lack bona fide emetic activity [29], which may result in fewer side effects. Also, SpeC is very well studied in terms of both structure [4], [30], [31] and function [9], [32]–[35] for engagement of host receptors, providing a platform for tailoring activity. Herein, we demonstrate that SpeC mutagenized within the zinc-dependent, high-affinity MHC-II binding domain (SpeC_D203A_) has reduced superantigenicity while retaining tumoricidal properties. We generated a SpeC_D203A_-based TTS fusion protein using an engineered human scFv that specifically targets human 5T4 (scFv5T4). In a humanized mouse model of colon cancer, we demonstrate that the scFv5T4::SpeC_D203A_ TTS controls the growth and metastatic potential of an established colon cancer tumor, and that this anti-tumor activity requires both specific targeting by the scFv5T4 moiety, as well as SAg function.

## Materials and Methods

### Ethics statements

Experiments using primary human lymphocytes were reviewed and approved by Western University's Research Ethics Board for Health Sciences Research Involving Human Subjects. Informed written consent was obtained from all blood donors. All animal experiments were in accordance with the Canadian Council on Animal Care Guide to the Care and Use of Experimental Animals, and the protocol was approved by the Animal Use Subcommittee at Western University (London, Ontario).

### Antibodies and dyes

The following monoclonal antibodies and dyes were used: PE anti-human CD4 (clone RPA-T4; BD Pharmingen); AlexaFluor700 anti-human CD8 (clone RPA-T8; BD Pharmingen); APC anti-human CD3 (Clone UCHT1; BD Pharmingen); CellTrace CFSE (carboxyfluorescein diacetate; Molecular Probes); 7-AAD (7-aminoactinomycin D; Molecular Probes); anti-human 5T4 (ab88091; Abcam); IgG2b isotype (eBioscience); FITC anti-mouse IgG (eBioscience); strepativdin-IRDye800 (Rockland Immunochemicals); streptavidin-FITC (Rockland Immunochemicals).

### Bacterial strains

*Escherichia coli* XL1-Blue (Stratagene) or DH5α (Invitrogen) were used for cloning purposes and *E. coli* BL21 (DE3) (Novagen) was used as the protein expression host. *E. coli* strains were grown aerobically at 37°C in Luria broth (LB) containing kanamycin (50 µg/ml), ampicillin (200 µg/ml) or chloramphenicol (10 µg/ml) to maintain plasmids.

### Cloning procedures

Plasmid constructs were either previously published [34], [35] or generated by standard cloning techniques [36], in either pET-41a (Novagen) or pET-32a (Novagen) and are summarized in **Table S1**. All plasmid inserts were sequenced at the Robarts Research Institute Sequencing Facility (London, Ontario, Canada). Protein expression clones in pET-32a or pET-41a were altered such that the enterokinase cleavage site (DDDDK↓X) was replaced with a Tobacco Etch Virus (TEV) protease cleavage site (ENLYFQ↓S). Transfection vectors pCMV6-XL5, pCMV6-XL5::5T4 and pEGFP-N1 were purchased from Origene Technologies, and Clonetech Laboratories, respectively. All other transfection plasmids were generated by standard cloning techniques. The murine scFv5T4 cDNA [37] was recoded and then manufactured by GenScript Inc. to generate a humanized sequence. Amino acid substitutions were made in the backbone sequence of scFv5T4 from the original mouse scFv sequence, determined by aligning with a human consensus sequence. The CDR loops specific for 5T4 [37], and the immediate amino acids flanking the predicted loops were not altered to maintain antibody specificity.

### Protein expression

Recombinant proteins were produced using an *E. coli* BL21 (DE3) expression system containing the pBirACm plasmid. Cells were grown aerobically at 37°C in LB medium to OD_600_ = 0.5 and protein expression was induced overnight (18–24 h) at room temperature (RT) with 0.2 mM isopropyl-D-thiogalactopyranoside (IPTG; BioBasic Inc.) and biotinylated with the addition of 50 µM D-biotin (BioBasic Inc.). Cells were pelleted at 4°C and resuspended in cold 20 mM Tris-HCl, pH 7.4, 200 mM NaCl containing 0.25 mg/ml lysozyme (Sigma-Aldrich) and 0.02 mg/ml DNase I (Sigma-Aldrich). Cells were incubated on ice for 1 h prior to lysis with a continuous head flow cell disruptor (Constant Systems Ltd.) at 25 psi, followed by sonication with output 4, 1 pulse/ml. Cellular debris was pelleted at 4°C at 10000×g. Supernatants were applied to a charged Ni-NTA affinity column (Novagen) and increasing concentration of imidazole was used to elute the purified protein. Purified fractions were dialyzed 3× against 20 mM Tris-HCl, pH 7.4, 200 mM NaCl buffer and the N-terminal tags were cleaved by autoinactivation-resistant His_7_::TEV [38], as described [39]. Cleaved proteins were applied and eluted from a second Ni-NTA affinity column to remove TEV protease and obtain a pure protein. Proteins were dialyzed 3× against 20 mM Tris-HCl, pH 7.4, 200 mM NaCl buffer or 0.9% NaCl (saline) and assessed for homogeneity by SDS-PAGE and quantified (BCA Protein Assay, Pierce).

### Cell lines

Human colorectal adenocarcinoma cell lines (HT-29 and WiDr) were cultured in complete Dulbecco's Modified Eagle Medium (cDMEM; Gibco) and HEK293 cells were cultured in complete Minimum Essential Media (cMEM; Gibco). All culture media were supplemented with 10% fetal bovine serum (FBS; Sigma-Aldrich), 10 mM HEPES, pH 7.4 (Gibco), 2 mM L-Glutamine (Gibco), 1 mM sodium pyruvate (Gibco), 100 µM non-essential amino acids (Gibco), 100 µg/ml streptomycin (Gibco), and 100 U/ml penicillin (Gibco).

### scFv5T4 specificity assays

HEK293 cells (1×10^5^) were seeded into 24-well plates (Corning) with 500 µl cMEM and allowed to grow overnight (24 h) at 37°C with 5% CO_2_. Liposome∶DNA complexes were formed using Lipofectamine2000 (Invitrogen) and plasmid DNA of choice as per the manufacture's protocol. Complexes were formed in cMEM without FBS or antibiotics. Transfection of cells occurred in the same media for 4 h at 37°C with 5% CO_2_, after which the media was removed and replaced with cMEM for plasmid expression over 24 h. Once expressed, scFv5T4::mRFP1 (1∶100; 2 mg/ml) was incubated with the cells for 1 h at RT and subsequently washed and viewed with fluorescence microscopy using an Olympus IX71 fluorescent microscope. Alternatively, transfected HEK293 cells (as above) or HT-29 cells (1.0×10^6^) were incubated for 1 h with mAb5T4 (1∶200) or scFv5T4-biotin (1∶100; 2 mg/ml), followed by anti-mouse IgG-FITC (1∶1000) or streptavidin-FITC (1∶1000), respectively, for 1 h at 4°C and viewed with fluorescence microscopy or FACS (BD FACSCanto II), respectively. Microscopy images were taken using ImagePro Plus Software, and FACS analysis was completed using Flowjo Software.

### Proliferation assays

Human peripheral blood mononuclear cells (PBMCs) were prepared from the whole blood of healthy donors and isolated by density centrifugation over Ficoll-Paque Plus (GE Healthcare Life Sciences). Human lymphocytes were cultured in RPMI-1640 (Gibco) with 10% FBS and supplemented as above (cRPMI). All tissue culture cells were maintained at 37°C with 5% CO_2_. Human PBMCs were labeled with CellTrace CFSE (Molecular Probes) as per manufacturer's instructions. Cells (0.8×10^6^–1.0×10^6^) were cultured in cRPMI containing 2 µg/ml Polymyxin B (ICN Biomedicals Inc.) and treated with either wild-type SpeC (SpeC_WT_) or variants SpeC_Y15A_, SpeC_D203A_ or SpeC_Y15A/D203A_ (1 µg/ml) and incubated for 5 days at 37°C with 5% CO_2_. Cells were then washed and stained with anti-human CD3 (1∶200), anti-human CD4 (1∶200) and anti-human CD8 (1∶200) antibodies for 30 min on ice and analyzed by FACS (BD Canto II), using FlowJo software. For radioactive proliferation assays, human PBMCs (2.0×10^5^) were cultured in cRPMI containing 2 µg/ml Polymyxin B (ICN Biomedicals Inc.) with titrating SpeC variants, scFv5T4, scFv5T4::SpeC_D203A_ or scFv5T4::SpeC_Y15A/D203A_ in U-bottom 96-well microtitre plates (BD Biosciences). Cells were incubated for 72 h and subsequently labeled with ^3^H-thymidine (Perkin Elmer Inc.) for 18 h at 37°C with 5% CO_2_. Cells were harvested onto glass-fibre filters and DNA-incorporated ^3^H-thymidine was measured in a beta scintillation counter (Wallac 1450 Microbeta Counter).

### Cytotoxicity assays

Two assays were used to measure the ability of the various proteins to induce PBMC-mediated killing of cancer cells. First, *in vitro* killing was evaluated by co-culturing human PBMCs with either WiDr cells or HT-29 at a ratio of 10∶1 and titrating SAgs including SpeC_WT_, SpeC_Y15A_, SpeC_D203A_, or SpeC_Y15A/D203A_ for 48 h. Cells were labeled with 7-AAD following the manufacturer's protocol and analyzed by FACS (BD Canto II). Using FlowJo software, the WiDr or HT-29 populations were gated upon by comparison of human PBMC alone samples and subsequently assessed for presence or absence of 7-AAD. Second, human PBMCs were treated with SAg, scFv5T4 or fusion proteins as in the FACS assay for 48 h in a U-bottom microtitre plate (BD Biosciences). Target HT-29 cells were labeled with (Na)_2_^51^CrO_4_ (Perkin Elmer Inc.) in cRPMI. PBMCs were added at effector∶target cell ratios of either 1∶1, 5∶1 or 10∶1 against HT-29. Cytotoxicity was measured after 4–6 h incubation at 37°C with 5% CO_2_ in a standard chromium release assay measuring the ^51^Cr content of culture supernatants using a gamma-counter (Wallac Wizard 1470 Automatic Counter). Total release control was obtained by exposing target cells to 1% sodium dodecyl sulfate (EMD Millipore). The specific lysis was calculated according to the formula: 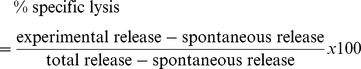



### Evaluation of tumor burden and metastases

Immunodeficient NOD.Cg-Prkdc^scid^ Il2rgt^m1Wjl^/SzJ (NSG) mice were bred in an animal barrier facility, and housed under sterile conditions with food and water ad libitum. Based on a previously developed protocol [18], [40], 13-week old mice were injected intraperitoneally with 3×10^6^ HT-29 cells in 0.2 mL vehicle (PBS). Three weeks later, after tumors were palpable, the mice were injected intraperitoneally with either vehicle alone (n = 3) or 1×10^6^ human PBMCs in 0.2 mL vehicle (n = 20). PBMC-treated mice were grouped (n = 4) with a random number generator to receive either scFv5T4::SpeC_D203A_, or controls SpeC_D203A_, scFv5T4, scFv5T4::SpeC_Y15A/D203A_, or vehicle alone. Two hours after receiving PBMCs, 2 µM/kg of treatment, controls, or vehicle alone was injected intravenously. Mice with no PBMCs received vehicle alone. Intravenous treatment injections were given daily for 7 additional days. After 4 weeks, the mice were sacrificed and the total tumor volume was determined. The mice were also examined visually for macro-metastases and scored accordingly based on the degree of regional spread distant from the primary tumor site. All tumors were excised, size- and weight-measured in a blinded fashion.

### Statistical analysis

Statistical comparisons were performed using an unpaired Student *t* test or by 2-way ANOVA with Bonferroni multiple comparison test (GraphPad Prism). Differences were considered significant when p<0.05.

## Results

### Generation of a SpeC-based TTS

**Figure 1:**
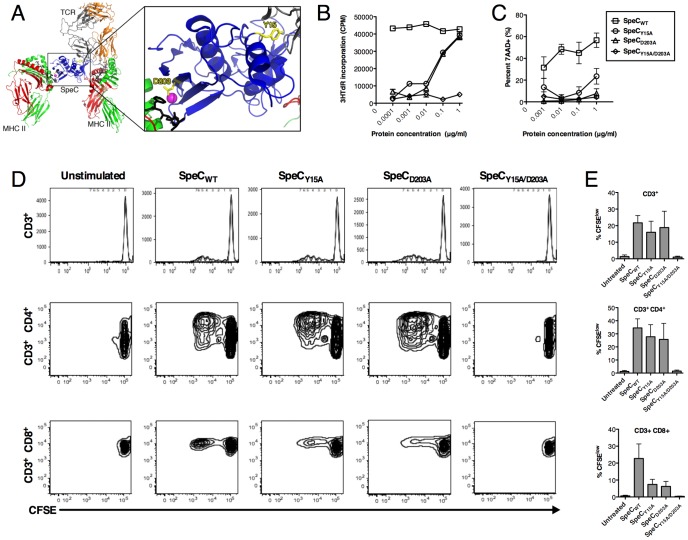
Overview of the SpeC-mediated T cell activation complex and mutations to reduce systemic toxicity. A) Structural overview of SpeC in complex with TCR and MHC-II. TCR Vα chain is colored orange, TCR Vβ chain is colored grey, MHCα-chains are colored red, MHCβ-chains are colored green, antigenic peptides are colored black, and the zinc atom is colored magenta. SpeC is colored blue with important interface residues Y15 and D203 highlighted in yellow. The ternary model of TCR-SpeC-(MHC)_2_ was produced as described previously [9] and the ribbon diagram was generated using PyMOL (http://www.pymol.org). B) Proliferation of human PBMCs mediated by SpeC_WT_ or proteins containing mutated residues Y15A (TCR-binding mutant), D203A (MHC-II-binding mutant) or Y15A/D203A was determined by the uptake of ^3^H-thymidine after 72 h post-stimulation (n = 5 in triplicate; data representative of one individual). C) Dose-dependent cytotoxicity of 7-AAD^+^ WiDr cells after 48 h incubation with human PBMCs and either SpeC_WT_, SpeC_Y15A_, SpeC_D203A_, or SpeC_Y15A/D203A_ (n = 3–6 per group). (D–E) Proliferation of CFSE labeled-human PBMCs mediated by SpeC_WT_ or proteins containing mutated residues was determined by FACS five days post-stimulation, specifically measuring total CD3^+^ T cell population, CD3^+^CD4^+^ T cells and, CD3^+^CD8^+^ T cells (n = 4; FACS data representative of one individual).

SpeC is a potent and well-characterized streptococcal SAg known to target primarily Vβ2^+^ human T cells [32] which represent ∼7% of the approximately 25 million distinct TCRs [41]. Prior work indicates that Tyr^15^ is a critical residue for this SAg to engage the TCR [34], and Asp^203^ is necessary to co-ordinate a zinc-mediated high-affinity interface with the β-chain of MHC-II [4], [35], [42] (**Figure 1A**). Indeed, the single Asp^203^→Ala mutation in SpeC has been demonstrated to dramatically reduce toxicity in a lethal model of toxic shock syndrome [42]. We first evaluated the ability of wild-type SpeC (SpeC_WT_), SpeC_Y15A_, SpeC_D203A_, and SpeC_Y15A/D203A_ to activate human PBMCs and induce PBMC-mediated killing of cancer cells. Both SpeC_Y15A_ and SpeC_D203A_ were impaired for the ability to expand PBMCS by ∼100-fold compared with SpeC_WT_, and the SpeC_Y15A/D203A_ double mutant was unable to induce PBMC proliferation (**Figure 1B**). Next, PBMC-dependent killing of the human colorectal cancer cell line WiDr was evaluated. SpeC_Y15A_ caused a significant reduction in WiDr cytotoxicity compared with SpeC_WT_, and both the SpeC_D203A_ and SpeC_Y15A/D203A_ mutants failed to induce WiDr cytotoxicity (**Figure 1C**). We also assessed the ability of the recombinant proteins to specifically induce proliferation of human CD3^+^CD4^+^ and CD3^+^CD8^+^ T cell populations at 1 µg/ml. SpeC_WT_ and each of the single mutants were able to induce proliferation of both subsets, while the double mutant failed to induce proliferation of either subset (**Figure 1D and 1E**). These data indicate that TCR and MHC-II engagement are important for induction of immune cell-mediated killing by SpeC_WT_, and that SpeC_D203A_ may be a suitable mutant to reduce or prevent systemic immune cell activation while maintaining full engagement with the TCR.

### Engineered human scFv5T4 specifically targets the 5T4 tumor-associated antigen

In order to develop a specific targeting mechanism for SpeC_D203A_, we generated a humanized scFv based on the complementarity determining regions (CDRs) of the characterized mouse scFv specific for the human 5T4 TAA [37]. The cDNA sequence was designed to incorporate a ‘humanized’ backbone sequence, with the CDRs remaining specific for human 5T4. Amino acid substitutions were determined by aligning the previously described mouse scFv5T4 with 10 human scFv sequences generating a consensus sequence. This cDNA sequence was codon optimized for *E. coli* and synthesized, and was subsequently used for the generation of a number of recombinant proteins (**Table S1**).

**Figure 2:**
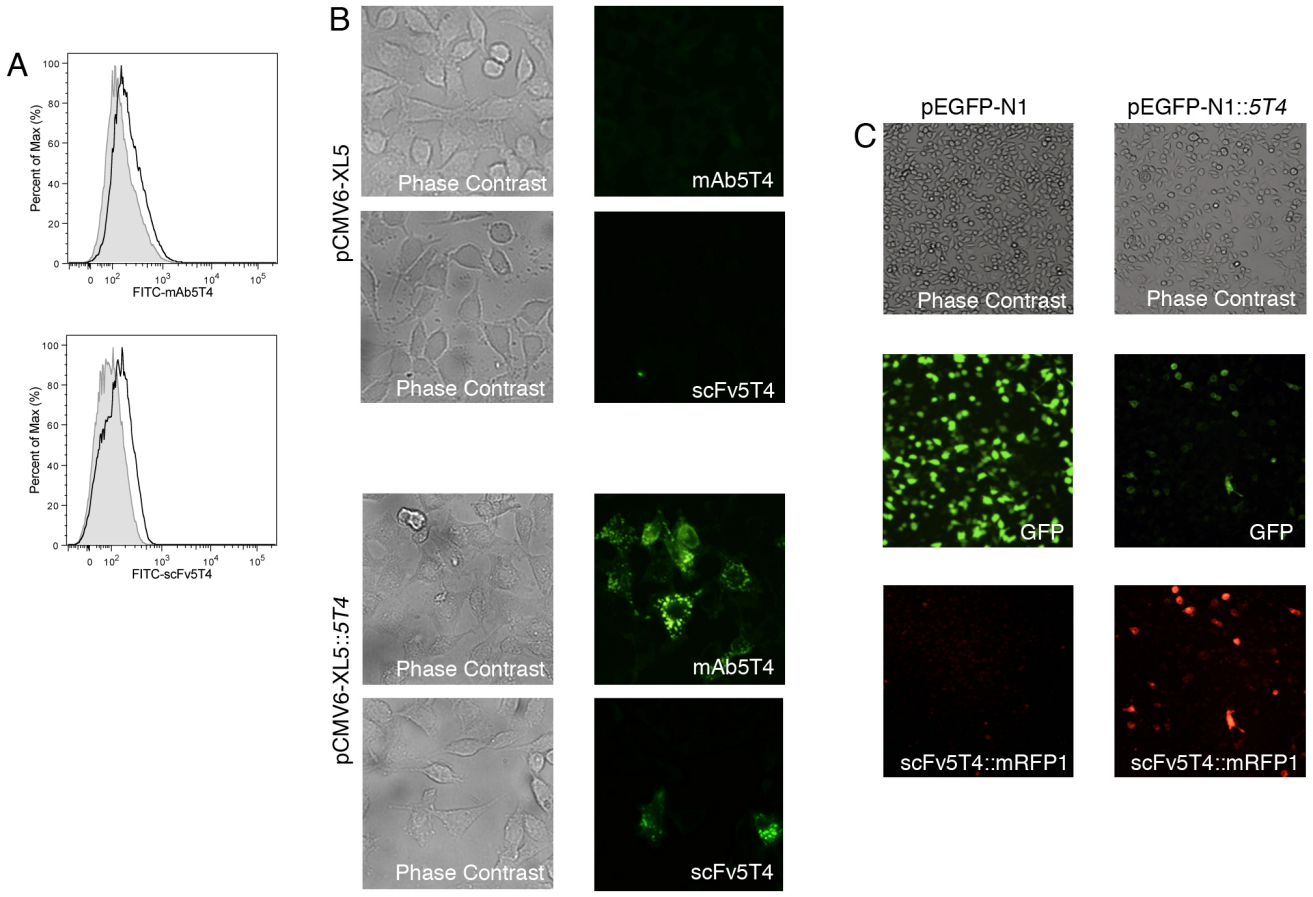
Specific targeting of scFv5T4. A) Histograms demonstrating surface binding of the indicated antibodies, either commercial mAb5T4 or generated scFv5T4 (empty curves), to 5T4 TAA on colorectal cancer cell line HT-29 measured by FACS. The shaded curves show the IgG2b isotype control (top panel) or streptavidin-FITC alone (bottom panel). B) Visualization of commercial mAb5T4, or scFv5T4, targeting of HEK293 cells transfected with empty vector (pCMV6-XL5) or pCMV6-XL5::5T4 by fluorescence microscopy. Representative images taken at 400× magnification. C) Visualization of HEK293 transfected with pEGFP-N1 or pEGFP-N1::5T4 and incubated with scFv5T4::mRFP1. Same field of view photographs were taken under phase contrast, and green and red fluorescent filters at 100× magnification.

To first examine the specificity of the humanized scFv5T4 for binding to human 5T4, the scFv5T4 cDNA was engineered to contain a C-terminal biotin tag, or genetically fused to monomeric red fluorescent protein 1 (mRFP1) [43]. Upon incubation with the human HT-29 colorectal cancer cells known to express 5T4 [44], from which WiDr cells are derived [45], scFv5T4 bound to the surface of these cells comparably to commercial anti-human 5T4 mAb (**Figure 2A**). HEK293 cells engineered to express the 5T4 antigen bound both the mAb5T4 as well as the scFv5T4 fragment as shown by immunofluorescence microscopy, whereas control HEK293 cells that contain only the vector did not stain with either antibody (**Figure 2B**). scFv5T4 specificity for 5T4 was also determined by incubation of scFv5T4::mRFP1 with HEK293 cells transfected with pEGFP-N1::5T4, or control vector pEGFP-N1. Microscopic analysis of GFP::5T4-expressing HEK293 cells demonstrated that scFv5T4 bound only to those cells expressing the 5T4::GFP fusion, but not to control transfected cells (**Figure 2C**). Together, these data indicate that the humanized scFv5T4 can bind specifically to human 5T4.

### Generation of scFv5T4::SpeC_D203A_

**Figure 3:**
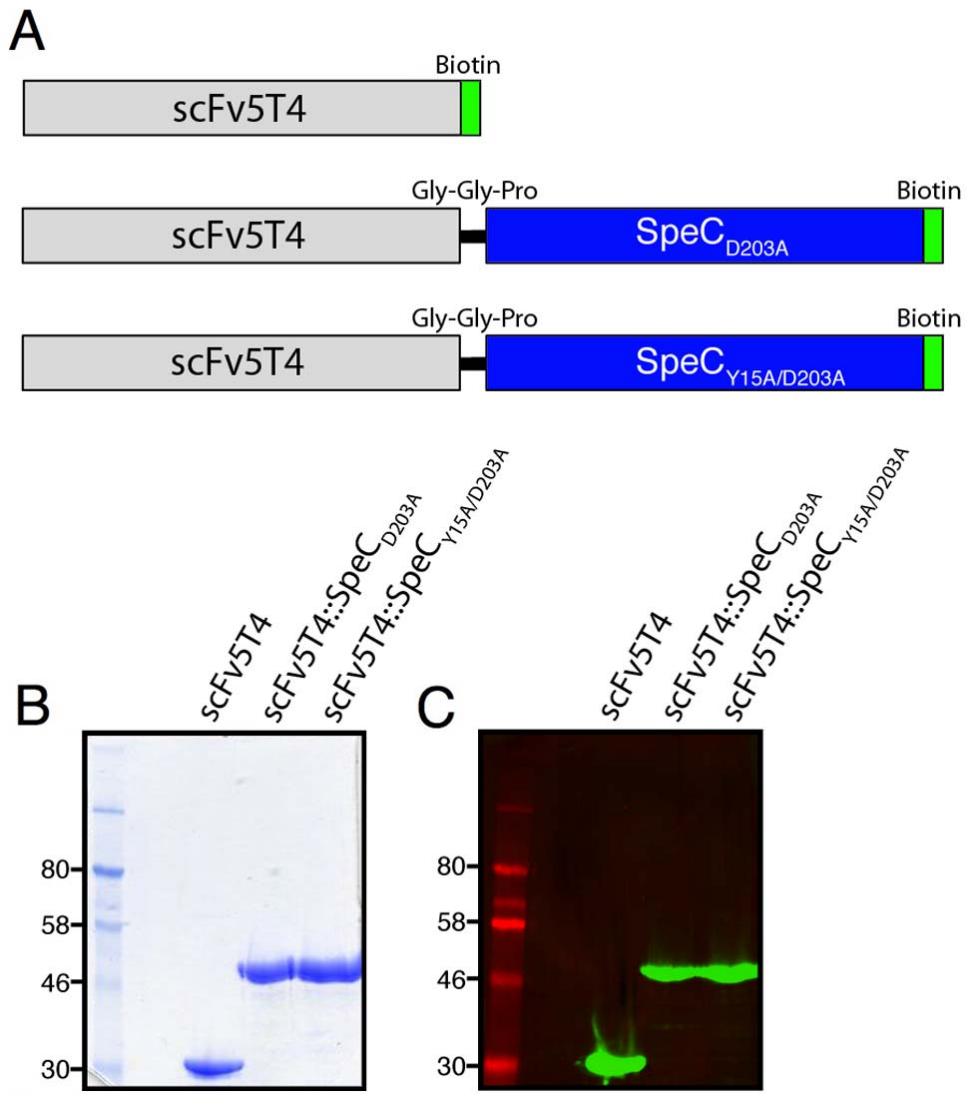
Generation of the scFv5T4::SpeC_D203A_ fusion protein and control reagents. A) Schematic illustration representing the components of the generated fusion protein constructs. The protein consists of the generated 5T4-targeted humanized single chain variable fragments, V_H_ and V_L_ (grey bar), genetically fused to streptococcal superantigen SpeC (blue bar) either containing an alanine substitution at residue D203 or an additional alanine substitution at residue Y15. All constructs were generated to contain a C-terminal biotin tag. The purified recombinant proteins are shown by SDS-PAGE (panel B), and detected by Western blot analysis by streptavidin-IRDye800 (panel C).

In order to target SpeC to 5T4, SpeC was translationally fused to scFv5T4 and recombinant scFv5T4::SpeC_D203A_ was expressed from *E. coli* BL21(DE3) and purified (**Figure 3**). In addition, control reagents were generated including scFv5T4 alone, and a non-functional fusion protein containing SpeC_Y15A/D203A_, each as soluble proteins containing C-terminal biotin tags (**Figure 3**).

### Human T-cell proliferation and cytotoxicity induced by scFv5T4::SpeC_D203A_

**Figure 4:**
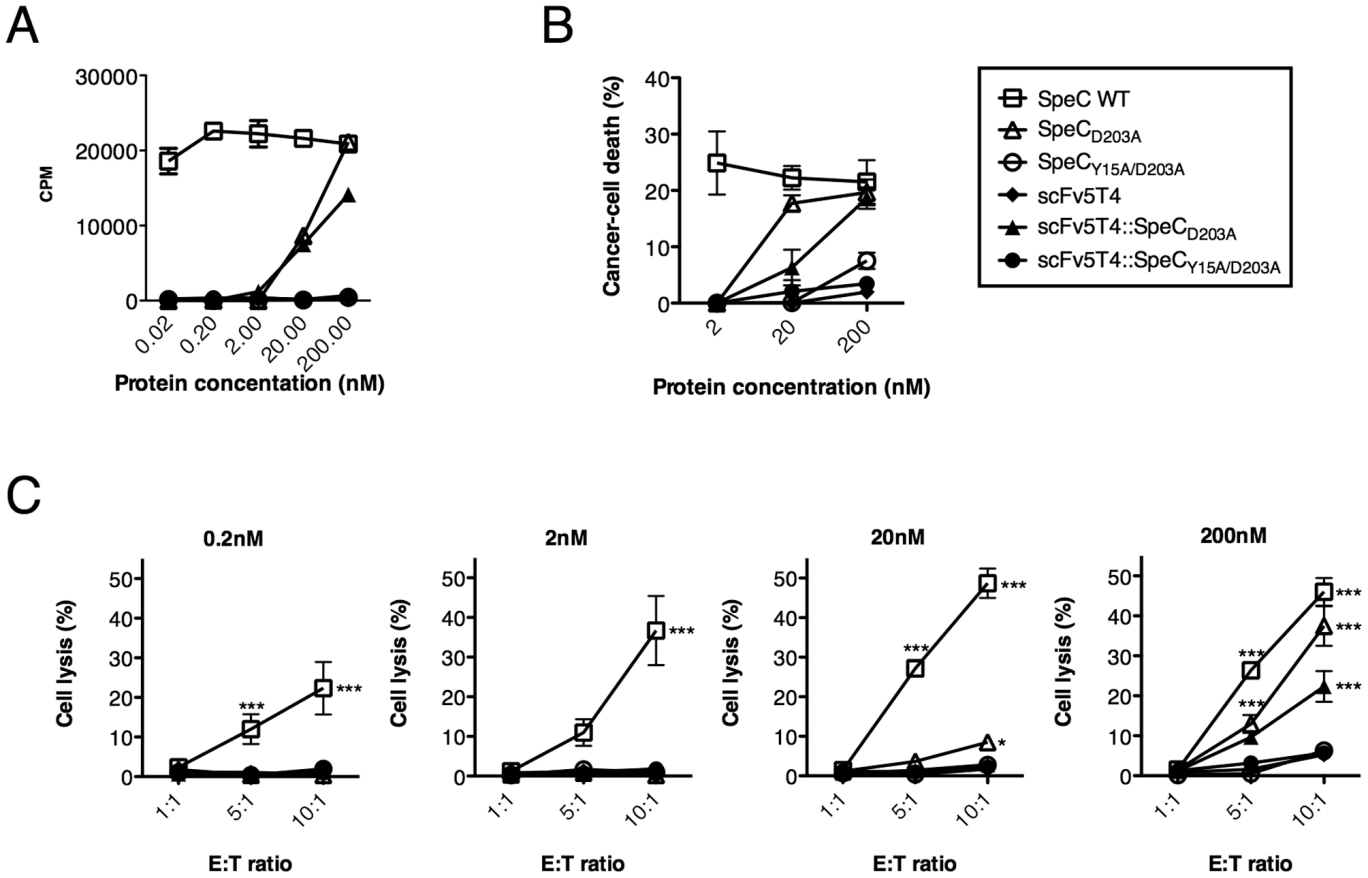
Functionality of SpeC mutants and fusion proteins for human PBMC proliferation and cytotoxicity *in vitro*. A) SpeC proteins were used to compare scFv5T4 alone, scFv5T4::SpeC_Y15A/D203A_ and subsequently scFv5T4::SpeC_D203A_ in the uptake of ^3^H-thymidine as a measure of PBMC proliferation after 4 day incubation (n = 5). B–C) Dose-dependent SpeC-mediated PBMC cytotoxicity of scFv5T4::SpeC_D203A_ was determined by comparing SpeC controls, scFv5T4 alone and scFv5T4::SpeC_Y15A/D203A_ after 48 h incubation by using FACS analysis of WiDr (panel B), measuring percent cancer cell death with 7AAD-exclusion staining (n = 3) and ^51^Cr-release to measure the specific cytotoxic potential (panel C) when incubated with increasing effector∶target ratios and ^51^Cr-labeled HT-29 cancer cells. Data shown (mean ±SEM) is from four independent human donors each done in triplicate. *p<0.05, ***p<0.001, compared to the inactive SpeC_Y15A/D203A_ control protein.

We first tested scFv5T4::SpeC_D203A_ and the control proteins for the ability to proliferate induced human PBMCs. scFv5T4::SpeC_D203A_ induced a dose-dependent proliferative response of human lymphocytes that was comparable to SpeC_D203A_ (**Figure 4A**). Importantly, the scFv5T4 antibody fragment alone and the double mutant fusion (scFv5T4::SpeC_Y15A/D203A_) did not induce significant proliferative responses. This indicates that the SpeC_D203A_ portion of the fusion is responsible for inducing PBMC activation. The immunotherapeutic agent was then evaluated for the ability to mediate tumor cell killing by human SpeC-reactive PBMCs in two assays. First, the human colorectal cancer cell line WiDr was used as the target in a 7-AAD-based killing assay. Efficient cell killing was observed after human PBMCs were stimulated with 200 nM of the agent for 48 hours, compared to wild-type SpeC and unstimulated controls (**Figure 4B**). Second, HT-29 cells labeled with ^51^Cr were used as targets. Efficient cell killing was observed in a dose-dependent manner after human PBMCs were stimulated with the agent for 48 hours, and subsequently added to tumor cells with increasing effector to target (E∶T) ratios (**Figure 4C**). Furthermore, the single mutant fusion (scFv5T4::SpeC_D203A_) was more efficient than that of the similar double mutant fusion (scFv5T4::SpeC_Y15A/D203A_) or antibody alone, but was reduced when compared with SpeC_WT_. These data indicate that scFv5T4::SpeC_D203A_ is functional for inducing immune cell-mediated cancer cell death and that SpeC_D203A_, scFv5T4, and scFv5T4::SpeC_Y15A/D203A_ proteins can function as precise controls to evaluate the requirement for targeting and SAg activity *in vivo*.

### Immunotherapy of established colon cancer using scFv5T4::SpeC_D203A_

**Figure 5:**
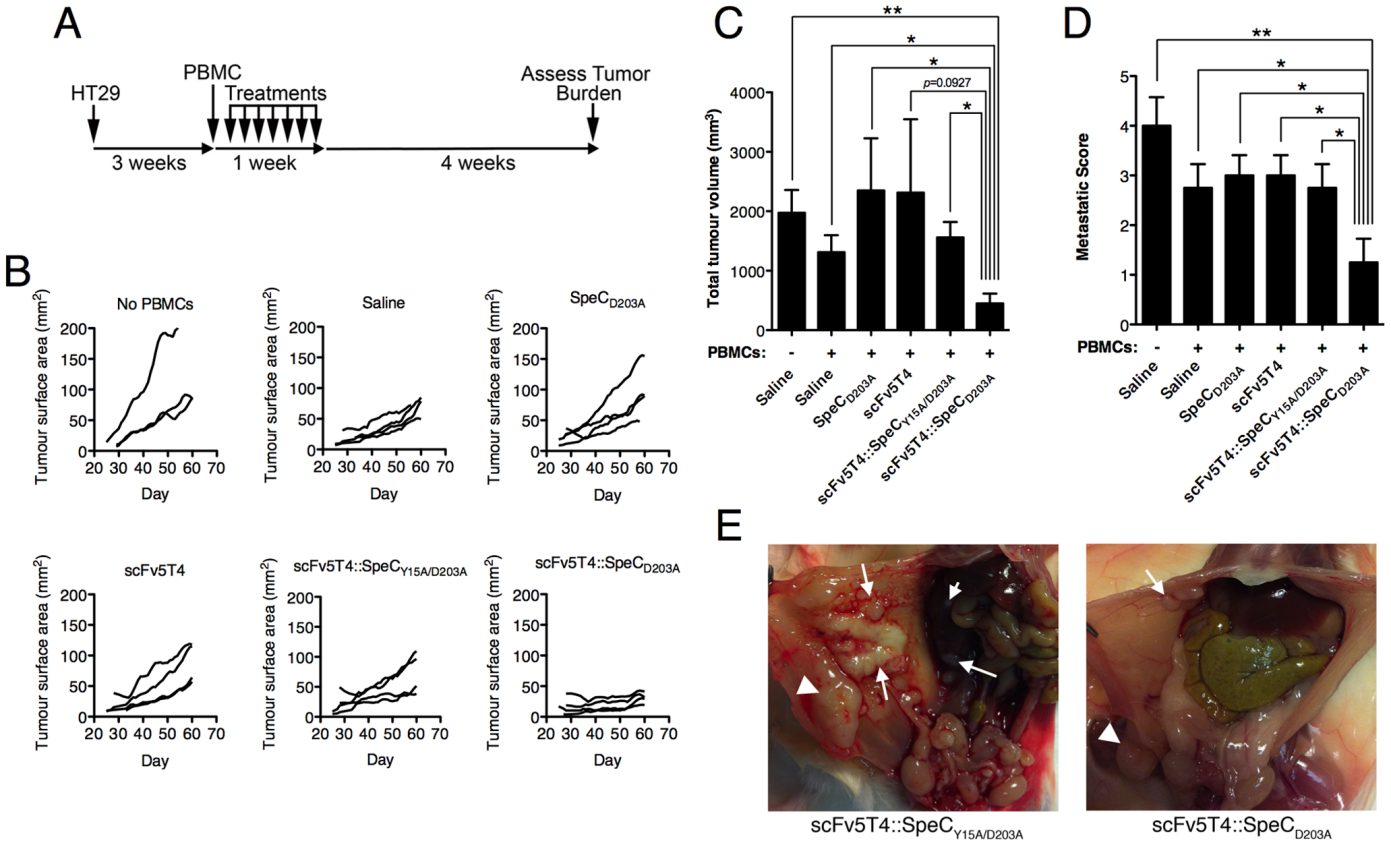
SpeC-based TTS therapy of established HT-29 colon cancer. A) Schematic illustration of the xenograft solid tumor model experimental timeline. NSG mice with established (3 week) intraperitoneal human HT-29 tumors were injected once with human PBMC intraperitoneally, followed by 8 daily intravenous injections of scFv5T4::SpeC_D203A_, or individual controls (2 µM/kg/injection). B) Primary tumor size was evaluated throughout the experimental timeline by external caliper measurements. Twenty-eight days post-final injection, final tumor volume was measured (panel C) and metastatic score (panel D) evaluated. All groups contained n = 4, with exception of saline alone control (n = 3). *p<0.05, **p<0.005. Gross pathology and metastases in representative NSG mice with HT-29 tumors treated with scFv5T4::SpeC_Y15A/D203A_ or scFv5T4::SpeC_D203A_. The primary tumor is labeled with a triangle and metastases are labeled with arrows.

SpeC is specific for human Vβ2^+^ T cells, but this SAg does not recognize mouse T cells [32]. Thus, testing the SpeC-based TTS required a model utilizing human lymphocytes. Furthermore, the human 5T4 targeting scFv has minimal cross-reactivity with murine 5T4 [37]. Therefore, human tumor cells expressing human 5T4 were necessary for the experiments. Based on a previously developed model [18], [40], we employed immunodeficient NOD SCID IL2Rγ^−/−^ (NSG) mice for the engraftment of 5T4^+^ human HT-29 colorectal adenocarcinoma cells. NSG mice lack T, B and NK cells [46] and represent an optimum mouse strain for human tumor engraftment [47]. Furthermore, these mice permit the survival of transferred human immune cells [46], [48]. HT-29 cells were injected intraperitoneally into NSG mice and once solid tumors were palpable (at 3 weeks post-injection), treatments were initiated with intraperitoneal injection of human PBMCs, followed by 8 daily intravenous injections of scFv5T4::SpeC_D203A_ (**Figure 5A**). Control NSG mice did not receive PBMCs, or received PBMCs without additional treatments. Additional groups included the scFv5T4 alone, SpeC_D203A_ alone, or inactive scFv5T4::SpeC_Y15A/D203A_. Tumor surface area was monitored using caliper measurement throughout the experiment and demonstrated little to no growth of the tumors in the scFv5T4::SpeC_D203A_ treatment group, while growth was observed in all other groups (**Figure 5B**). Mice were sacrificed at week 8 of the experiment and tumors were evaluated in a blinded fashion. This experiment demonstrated a dramatic reduction in the total tumor volume after treatment with scFv5T4::SpeC_D203A_ that was significantly different from mice that did not received PBMCs, sham treated mice (saline), and mice treated SpeC_D203A_ or scFv5T4::SpeC_Y15A/D203A_ (**Figure 5C**). Importantly, the scFv5T4::SpeC_D203A_ treatment group also demonstrated a significant reduction in the total metastases score compared with all other groups (**Figure 5D and 5E**). There were no differences in tumor volumes or number of metastases between mice that did not receive PBMCs and the different control reagents (**Figure 5D**).

## Discussion

T lymphocytes are recognized as one of the most important immune cells involved in tumor regression in cancer immunotherapy, and bacterial SAgs are among the most potent naturally occurring specific activators of T cells. Thus, the appropriation of SAgs to target cancer cells [17] has received significant attention, and TTS therapeutics have now been evaluated in human clinical trials [22]–[24], [26], [49], [50].

In the current study, we describe the development of a “next generation” TTS composed of the streptococcal T cell activating toxin SpeC and a humanized scFv targeting the 5T4 TAA. In this work, we focused on colorectal cancer as this carcinoma is difficult to diagnose with few symptoms until the onset of stage III or IV, and ∼20% of patients will present with inoperable colorectal cancer [51]. The expression of the 5T4 TAA is restricted on normal adult tissues but is found on an array of carcinomas [52] and has been associated with metastasis in colorectal cancer [53]. This work provides further preclinical evidence for 5T4 as a potential TAA for targeted colorectal cancer immunotherapy, and that TTSs may be useful to inhibit or prevent further metastatic disease. As monoclonal antibodies that target vascular endothelial growth factor (VEGF) (e.g. Bevacizumab) and epidermal growth factor receptor (EGFR) (e.g. Cetuximab or Panitumumab) have shown benefit in patients with metastatic colorectal cancer [54]–[56], a future area of interest would be to evaluate TTS combination therapies with these more established treatments.

This work demonstrated that the soluble recombinant fusion protein scFv5T4::SpeC_D203A_ was able to specifically target 5T4 to elicit a T cell response that substantially reduced tumor burden *in vivo*. Importantly, we used a model of large and established tumors in order to robustly test the SpeC-based TTS. Although the tumors did not appear to regress, the data clearly demonstrates that scFv5T4::SpeC_D203A_ was able to prevent further tumor growth as well as the development of peritoneal metastases. As scFv5T4::SpeC_D203A_ and SpeC_D203A_ showed similar activity *in vitro* (**Figure 4a**), the inability of SpeC_D203A_ to impact tumor size or metastatic disease indicates that the scFv5T4 moiety of the fusion protein was required for *in vivo* targeting of 5T4^+^ HT-29 cells (**Figures 2A** **,** **5C and 5D**). Likewise, the inability of scFv5T4 alone, or the inactive scFv5T4::SpeC_Y15A/D203A_ fusion to show any measurable impact (**Figure 5C and 5D**) demonstrates that T cell-dependent SAg activity was also required for tumor cell killing. Although the SpeC_D203A_ mutant was designed to reduce systemic T cell activation, the mouse Fab moiety in the 5T4Fab-SEA/E-120 TTS has been shown to effectively replace the MHC-II binding domain such that T cells are efficiently activated when artificially ‘presented’ by the tumor [57]. We suspect that the humanized scFv5T4 moiety here played a similar role contributing to the dramatic reduction in tumor volume and metastatic disease.

There are some potential advantages, and disadvantages, in using TTSs for tumor immunotherapy that require further consideration. The use of a mouse derived antibody as a targeting motif may result in human anti-mouse antibody (HAMA) responses since murine mAbs are highly immunogenic [58]. This may limit the utility of subsequent treatments and thus the use of a humanized scFv containing TTS as developed in this work may provide clinical benefit. Second, bacterial SAgs are produced by bacteria that are often frequent colonizers in humans and thus many individuals will have pre-existing and neutralizing antibodies to many streptococcal and staphylococcal SAgs [59], [60]. To overcome this issue, we foresee the future generation of ‘combinatorial’ TTSs with different SAgs such that individual patients could be screened for SAg neutralizing antibodies and then treated with an appropriate TTS. Early work in this area demonstrated that multiple SEs are capable of inducing T cell-mediated cytotoxicity against cancer cells [61]; however, we envision the SAg panel would include members from the Group IV and Group V subclass of SAgs [1], [39], [62], as these subclasses contain only streptococcal SAgs and staphylococcal enterotoxin-like (SEl) SAgs, that collectively lack the emetic properties of the bona fide SEs [62]. Indeed, the SEl-M, SEl-N and SEl-O SAgs from the staphylococcal ‘enterotoxin gene cluster’ (*egc*) have recently been demonstrated to induce T cell dependent killing of a broad panel of human tumor cells *in vitro* [63]. Also, human serum levels of neutralizing antibodies against the *egc* SEs have been shown to be lower than those directed against the ‘classic’ SEs [64]. In addition, each of the Group IV and V SAgs have a well defined zinc-binding motif [1] involved in high-affinity MHC-II binding [4], [5] that can be targeted for appropriate mutagenesis to prevent systemic immune activation as shown here and previously for SEA [65]. A third important limitation to TTS immunotherapy is that bacterial SAgs are well known to induce Vβ-specific T cell deletion or anergy [66], which includes CD8^+^ T cells [67]. Thus, repeated administration of the same TTS in humans may result in populations of non-responsive T cells. However, using the B16 model of melanoma, a sufficient resting period between treatments did restore immune responsiveness resulting in prolonged survival with repeated cycles of therapy [68]. Nevertheless, this limitation could potentially be circumvented by the use of multiple SAgs with different Vβ profiles. A remaining and important issue with TTS immunotherapy is the targeting of a single TAA. An effective TTS would likely invoke a form of cancer immunoediting [69], and simple down regulation of the TAA may provide a means of escape. The TTS immunotherapy platform offers an approach for targeting a number of different TAAs, and it will be of future interest to engineer and combine TTSs that utilize SAgs with different Vβ profiles.

Immunotherapies such as the chimeric antigen receptors (CARs) targeting CD19 have now demonstrated some extraordinary clinical outcomes in patients with advanced B cell leukemia [70]–[73]. In addition, blocking immune system regulatory checkpoints with antibodies (e.g. anti-CTLA-4 or anti-PD-1) is providing additional avenues for cancer immunotherapy [74]. TTSs may represent a additional ‘off-the-shelf’ therapy to harness Vβ-specific T cell subsets without the requirement for manipulation of autologous T cells. This work may help to guide the ‘next generation’ of TTSs for tailored cancer immunotherapy.

## Supporting Information

Table S1 **Plasmids used in this study.**
(PDF)
