# Supplementary material for: Control of Established Colon Cancer Xenografts Using a Novel Humanized Single Chain Antibody-Streptococcal Superantigen Fusion Protein Targeting the 5T4 Oncofetal Antigen
Source: PLoS One. 2014 Apr 15;9(4):e95200. doi: 10.1371/journal.pone.0095200 (PMC3988171; doi:10.1371/journal.pone.0095200)
Supplement: Table S1 — Plasmids used in this study. (PDF) [file pone.0095200.s001.pdf]

Table S1. Plasmids used in this study.

Plasmid name	Relevant characteristics [a]	Source
pET-41a(+)	Protein expression vector; Km^r	Novagen
pET-41a(+)::TEV	Protein expression vector with modified protease cleavage site; Km^r	This study
pET-32a (+)	Protein expression vector; Ap^r	Novagen
pET-32a (+)::TEV	Protein expression vector with modified protease cleavage site; Ap^r	This study
pBirACm	pACYC184 with inducible biotin ligase; Cm^r	Avidity
pET-41a::TEV::*speC*$_{WT}$::biotin	*speC* inserted into *Nco*I and *Bam*HI of pET-41a with a C-terminal biotin tag; Km^r	This study
pET-41a::TEV::*speC*$_{Y15A}$	*speC*$_{Y15A}$ inserted into *Nco*I and *Bam*HI sites of pET-41a; Km^r	[34]
pET-41a::TEV::*speC*$_{D203A}$	*speC*$_{D203A}$ inserted into *Nco*I and *Bam*HI sites of pET-41a; Km^r	[35]
pET-41a::TEV::*speC*$_{Y15A/\ D203A}$	*speC*$_{Y15A/D203A}$ inserted into *Nco*I and *Bam*HI sites of pET-41a; Km^r	This study
pUC57::*scFv5T4*::biotin	Codon optimized scFv5T4 sequence with *Nco*I and *Hin*dIII sites; C-terminal biotin tag; Ap^r	GenScript
pET-32a::*scFv5T4*::*mRFP1*	*scFv5T4* from pUC57::scFv5T4 inserted into *Nco*I and *Hin*dIII sites of pET-32a; *mRFP1* inserted into *Eco*RI and *Hin*dIII sites replacing biotin tag of scFv5T4; Ap^r	This study
pET-32a::TEV::*scFv5T4*::biotin tag	*scFv5T4* from pUC57::*scFv5T4* inserted into *Nco*I and *Hin*dIII sites of pET-32a; Ap^r	This study
pET-32a::TEV::*scFv5T4*::*speC*$_{D203A}$::biotin	*scFv5T4* from pUC57::*scFv5T4* inserted into *Nco*I and *Hin*dIII sites of pET-32a; translationally fused with *speC*$_{D203A}$ with an engineered gly-gly-pro (GGP) linker; C-terminal biotin tag; Ap^r	This study
pET-32a::TEV::*scFv5T4*::*speC*$_{Y15A/D203A}$::biotin	*scFv5T4* from pUC57::*scFv5T4* inserted into *Nco*I and *Hin*dIII sites of pET-32a; translationally fused with speC$_{Y15A/D203A}$ with an engineered gly-gly-pro (GGP) linker; C-terminal biotin tag; Ap^r	This study
pCMV6-XL5	Mammalian expression vector; Ap^r	Origene
pCMV6-XL5::*egfp*	*egfp* from pEGFP-N1 inserted into *Sac*I and *Xba*I sites of pCMV6-XL5; Ap^r	This study
pCMV6-XL5::*5t4*	5T4 inserted into *Eco*RI and *Sal*I sites of pCMV6-XL5; Ap^r	Origene
pEGFP-N1	Mammalian expression vector with N-terminal EGFP tag; Km^r	Clontech
pEGFP-N1::*5t4*	5t4 from pCMV6-XL5::5t4 inserted into *Eco*RI and *Bam*HI sites of pEGFP-N1; Km^r	This study

[a] Kn^r, Kanamycin resistance; Ap^r, Ampicillin resistance; Cm^r, Chloramphenicol resistance
